# The Contribution of Genetic Variation and Aberrant Methylation of Aryl Hydrocarbon Receptor Signaling Pathway Genes to Rheumatoid Arthritis

**DOI:** 10.3389/fimmu.2022.823863

**Published:** 2022-03-02

**Authors:** Tian-Ping Zhang, Rui Li, Hong-Miao Li, Nan Xiang, Zhen Tan, Guo-Sheng Wang, Xiao-Mei Li

**Affiliations:** ^1^ Department of Rheumatology and Immunology, The First Affiliated Hospital of USTC, Division of Life Sciences and Medicine, University of Science and Technology of China, Hefei, China; ^2^ Department of Nosocomial Infection Management, The First Affiliated Hospital of Anhui Medical University, Hefei, China; ^3^ Department of Epidemiology and Biostatistics, School of Public Health, Anhui Medical University, Anhui Provincial Laboratory of Inflammatory and Immune Diseases, Hefei, China

**Keywords:** autoimmune disease, aryl hydrocarbon receptor, methylation, single nucleotide polymorphisms, rheumatoid arthritis

## Abstract

The aryl hydrocarbon receptor (AHR) signaling pathway participates in immune regulation of multiple autoimmune diseases, including rheumatoid arthritis (RA). We conducted this study to investigate the association of AHR signaling pathway genes (*AHR*, *ARNT*, *AHRR*) single nucleotide polymorphisms (SNPs), as well as their methylation levels, with RA susceptibility. Nine SNPs (*AHR* gene rs2066853, rs2158041, rs2282885, *ARNT* gene rs10847, rs1889740, rs11204735, *AHRR* gene rs2292596, rs2672725, rs349583) were genotyped *via* improved multiple ligase detection reaction (iMLDR) in 479 RA patients and 496 healthy controls. We used the Illumina Hiseq platform to detect methylation levels of these genes in 122 RA patients and 123 healthy controls. A significant increase in rs11204735 C allele frequency was observed in RA patients when compared to controls. Further, rs11204735 polymorphism was associated with a decreased risk of RA under the dominant model. *ARNT* CCC haplotype frequency was significantly increased in RA patients in comparison to controls. In the *AHRR* gene, rs2672725 GG genotype, G allele frequencies were significantly related to an increased risk of RA and rs2292596, rs2672725 polymorphism were significantly associated with an increased risk of RA under the dominant model, recessive model, respectively. However, no significant association was identified between *AHR* gene polymorphism and RA susceptibility. The *AHR* methylation level in RA patients was significantly higher than the controls, while *AHRR* methylation level was abnormally reduced in RA patients. In addition, *AHRR* rs2672725 genotype distribution was significantly associated with the *AHRR* methylation level among RA patients. In summary, *ARNT* rs11204735, *AHRR* rs2292596, and rs2672725 polymorphisms were associated with RA susceptibility and altered *AHR*, *AHRR* methylation levels were related to the risk of RA.

## Introduction

Rheumatoid arthritis (RA) is a common, chronic autoimmune disease that affects approximately 1% of the general population (1). The main clinical features of RA patients are synovial inflammation, bone and cartilage erosion, and symmetric polyarthritis with joint swelling (2), however, the pathogenesis of this disease is not fully understood. In previous studies, the immunological dysfunctions of RA were identified in various immune cells, such as T cells, B cells, macrophages, and abnormal expression levels of inflammatory cytokines, including IL-1, IL-6, IL-10, TNF-α, were observed in RA patients (3). In addition to immunological findings, many studies have also found an influence of genomic and epigenomic features, and environmental factors on disease activity, prognosis, and therapy prediction of RA (4–6). Genome wide association study and candidate gene studies have identified a large number of RA related genes/loci in different ethnic groups, however, these genes/loci only account for a small part of RA phenotypic variation (7, 8). DNA methylation is a widely studied form of epigenetic modification, and some prior studies have suggested that an association between DNA methylation and inflammation-regulated immune pathways may play an important role in the pathogenesis of RA (9).

Previous studies confirmed that environmental pollutants from smoking and hydrocarbon burning were associated with RA disease risk, as both pollutants contained agonists or exogenous ligands for aryl hydrocarbon receptor (AHR) that may promote AHR activation (10). As a ligand-activated transcription factor, AHR is involved in regulating the gene activity in response to hydrophobic halogenated aromatic hydrocarbons in order to promote the metabolism, and elimination of xenobiotics. The role of AHR in cellular responses against a variety of endogenous and physiological ligands have been extensively studied, while increasing evidence indicates that AHR is involved in the regulation of innate and adaptive immune responses, and immunologic processes in autoimmune disease, including multiple sclerosis (MS) (11, 12). For example, AHR expression is abnormally elevated in synovial tissues of RA patients and could regulate the expression of cytokines such as growth factors (13). Over-activation of AHR signaling may promote inflammation and bone destruction in RA by activating macrophages, osteoclasts, dendritic cells, and inhibiting osteoblasts (10).

In the regulation of the AHR signaling pathway, ligand binding to AHR causes conformational changes that induce this receptor to migrate to the nucleus where it can generate a hetero dimer with AHR nuclear translocation protein (ARNT) to form aryl hydrocarbon receptor complex, inducing the transcription of relevant target genes (14). In addition, AHR repressor (AHRR) serves as a negative regulator within this pathway through inhibiting AHR-mediated signal transduction (15). Recent studies have investigated the association of common, potentially functional single nucleotide polymorphisms (SNPs) in AHR signaling pathway genes with MS, essential hypertension (14, 16). Given the importance of the AHR signaling pathway in the development of RA, this present study aimed to evaluate the association between SNPs in the AHR signaling pathway genes (*AHR*, *ARNT*, *AHRR*) and RA susceptibility. The methylation levels of these genes were also detected in these patients because DNA methylation is considered to be an important factor for RA.

## Materials and Methods

### Patients and Healthy Controls

In this study, the participant included 479 RA patients and 496 healthy controls that were recruited to explore the association between *AHR*, *ARNT*, *AHRR* genes SNP and RA susceptibility. Of which 122 RA patients and 123 healthy controls were selected to simultaneously detect DNA methylation levels of *AHR*, *ARNT*, *AHRR* genes (**Table 1**). All patients were diagnosed by experienced clinicians according to the 1987 American College of Rheumatology revised criteria (17), and patients with other autoimmune diseases, infectious diseases, tumors were excluded from this study. Volunteers with no history of inflammatory/autoimmune diseases, cancers, *etc.* were recruited as healthy controls. The DNA samples, demographic data of all subjects were obtained from Anhui Provincial Laboratory of Inflammatory and Immune Diseases, and the clinical data of RA patients including anti-cyclic citrullinate peptide (anti-CCP) and rheumatoid factor (RF) was also recorded.

**Table 1:** The demographic and clinical characteristics of RA patients and healthy controls.

Characteristics	RA patients	Healthy controls
Genotyping		
Age (years)	52.71 ± 12.42	50.61 ± 14.76
Sex (male/female)	88 /391	112/384
anti-CCP-positive, n (%)	353 (73.70)	NA
RF-positive, n (%)	380 (79.33)	NA
Methylation test		
Age (years)	52.61 ± 13.05	46.93 ± 14.29
Sex (male/female)	22/100	41/82
anti-CCP-positive, n (%)	88 (72.13)	NA
RF-positive, n (%)	99 (81.15)	NA

n, number; NA, not applicable.

### SNP Selection and Genotyping

First, we used the public database (CHBS_1000G, Ensembl Genome Browser 85) to obtain the genotype data of the Han population in Beijing, China. Then, we screened tag SNPs capturing all the common SNPs located in the chromosomal locations of AHR signaling pathway genes (*AHR*, *ARNT*, *AHRR*) and the 2.0 kbp region of their flank. The selection was conducted using the Haploview 4.0 software (Cambridge, MA, USA). Next, we searched for the SNP in *AHR*, *ARNT* and *AHRR* genes that were significantly associated with other diseases, and other potentially functional SNP. Finally, our study selected three tagSNPs (rs2066853, rs2158041, rs2282885) in *AHR*, three tagSNPs (rs10847, rs1889740, rs11204735) in *ARNT*, three tagSNPs (rs2292596, rs2672725, rs349583) in *AHRR* for genotyping. All SNPs were consistent with MAF ≥ 0.05 in CHB and *r^2^* threshold > 0.8.

Genotyping was performed with improved multiple ligase detection reaction (iMLDR) under the technical support from the Center for Genetic & Genomic Analysis, Genesky Biotechnologies (Inc., Shanghai, China) (18). The detailed experimental steps were as follows: (1) 1μl DNA sample was extracted, and the quality of the sample was checked and the concentration was estimated by 1% agarose electrophoresis. Then the DNA sample was diluted to 5-10 ng/μl according to the estimated concentration. (2) Multiplex PCR reaction was carried out with 20μl reaction system included 1x HotStarTaq bufer, 3.0 mMMg2+, 0.3mM dNTP, 1U HotStarTaq polymerase (Qiagen Inc.), 1μl sample DNA and 1μl multiple PCR primers. (3) Purifcation of multiple PCR products: 5U SAP enzyme and 2U Exonuclease I enzyme were added to 20 μl PCR product, 37°C warm bath for 1h, then 75°C inactivated for 15min. (4) Ligating reaction system: 1 μl 10x ligating bufer, 0.25 μl high temperature ligase, 0.4 μl 5’ ligating primer mixture (1μM), 0.4 μl primer 3’ ligating primer mixture (2 μM), 2 μl purified multiple PCR products, 6 μl ddH2O mixing. (5) The 0.5 μl diluted product was mixed with 0.5 μl Liz500 SIZE STANDARD, 9 μl Hi-Di, denatured at 95°C for 5 min, then placed on the ABI3730XL sequencer. (6) The raw data collected on the ABI3730XL sequencer are analyzed by GeneMapper 4.1 (AppliedBiosystems, USA). The subjects with 100% genotyping success rate for all SNPs were included for final analysis.

The methylation levels of *AHR*, *ARNT*, *AHRR* genes were detected by MethylTarget^®^ with technical support from the Center for Genetic & Genomic Anal-ysis, Genesky Biotechnologies (Inc., Shanghai, China) (19). We sequenced the CpG islands in the promoter region of *AHR*, *ARNT*, *AHRR* genes using the Illumina Hiseq platform. Primers were designed to amplify the specific sites of interest from the bisulfite-converted DNA **(** **Table S1** **)**, and the mean methylation level of all CpG sites on the fragment was calculated as the methylation level of the specific sites of each gene.

The detailed experimental steps were as follows: (1) Genomic DNA (400ng) was subjected to sodium bisulfite treatment using EZ DNA Methylation™-GOLD Kit (Zymo Research) according to manufacturer’s protocols. (2) 2 μl bisulfite DNA was prepared for multiplex PCR reaction in 20 μl reaction mixture, included 1x reaction buffer (Takara), 1 U HotStarTaq polymerase (Takara), 3 mM Mg2+, 0.2 mM dNTP, and 0.1 µM of each primer for PCR amplification. The cycling program was as follows: 95°C for 2 min; 11 cycles of 94°C for 20 s, 63°C for 40s with a decreasing temperature step of 0.5°C per cycle, and 72°C for 1 min; subsequently 24 cycles of 94°C for 20 s, 65°C for 30 s, and 72°C for 1 min; and 72°C for 2 min. (3) 1 μl diluted PCR amplicons were used for index PCR reaction in 20 μl mixture, containing 1x reaction buffer (NEB Q5™), 1 U Q5™ DNA polymerase (NEB), 0.3 mM dNTP, 0.3 mM of F primer, and 0.3 μM of index primer. The cycling program was 98°C for 30 s, 11 cycles of 98°C for 10 s, 65°C for 30 s, and 72°C for 30 s; and 72°C for 5 min. (4) PCR amplicons (170bp-270bp) were separated by agarose electrophoresis and purified using QIAquick Gel Extraction kit (QIAGEN) and then loaded onto Illumina NextSeq 500 (Illumina, San Diego, CA, USA) according to the manufacturer’s protocols.

### Statistical Analysis

Statistical analysis was conducted with the SPSS 23.0 (Armonk, NY: IBM Corp, USA). Hardy–Weinberg equilibrium (HWE) was calculated in RA patients and healthy controls by using Chi-square (*χ^2^*). Genotype, allele distribution frequencies were compared using logistic regression analysis, and two genetic models (dominant, recessive) were also tested. The following statistical indexes, including *P* values, odds ratios (*OR*), and 95% confidence intervals (CI), were analyzed as measures of associations. Haplotype analysis for each gene was calculated using the SHEsis software. The methylation levels of candidate genes were expressed as mean and standard deviation, and the differences of methylation levels between two groups and three groups were conducted using T-test and variance analysis, respectively. *P* values < 0.05 were considered as statistically significant for all tests. The Bonferroni correction was used for multiple testing.

## Results

In this study, the mean age of the 479 RA patients was 52.71 ± 12.42 years, including 391 females and 88 males, and the average age of the 496 healthy controls was 50.61 ± 14.76 years, including 384 females and 112 males. The genotype distributions of all SNPs in RA patients were in accordance with HWE (rs2066853, *P* = 0.997; rs2158041, *P* = 0.792; rs2282885, *P* = 0.555; rs10847, *P* = 0.747; rs1889740, *P* = 0.530; rs11204735, *P* = 0.914; rs2292596, *P* = 0.713; rs2672725, *P* = 0.346; rs349583, *P* = 0.913). In healthy controls, *AHR* rs2158041 did not conform to HWE (*P* = 0.023), while other SNPs conformed to HWE (rs2066853, *P* = 0.249; rs2282885, *P* = 0.401; rs10847, *P* = 0.871; rs1889740, *P* = 0.461; rs11204735, *P* = 0.603; rs2292596, *P* = 0.373; rs2672725, *P* = 0.918; rs349583, *P* = 0.855). Therefore, this study excluded rs2158041, and the allele and genotype frequency distributions of other SNPs in RA patients and healthy controls were shown in **Tables 2**, **3**.

**Table 2:** Genotype frequencies of *AHR, ARNT, AHRR* genes in RA patients and controls.

SNP	Analyze model		RA patients	Controls	*P* value	*OR* (95% *CI*)
*AHR*					
rs2066853	Genotype	AA	68 (14.20)	63 (12.70)	0.649	0.912 (0.612,1.358)
		GA	225 (46.97)	244 (49.19)	0.639	1.067 (0.813,1.400)
		GG	186 (38.83)	189 (38.10)	Reference
	Dominant model	GG	186 (38.83)	189 (38.10)	0.816	1.019 (0.869,1.194)
		GA+AA	293 (61.17)	307 (61.90)	Reference
	Recessive model	AA	68 (14.20)	63 (12.70)	0.494	0.983 (0.935,1.033)
		GA+GG	411 (85.80)	433 (87.30)	Reference
rs2282885	Genotype	GG	25 (5.22)	24 (4.84)	0.964	0.987 (0.551,1.768)
		GA	159 (33.19)	185 (37.30)	0.189	1.196 (0.551,1.768)
		AA	295 (61.59)	287 (57.86)	Reference
	Dominant model	AA	295 (61.59)	287 (57.86)	0.236	1.064 (0.960,1.180)
		GA+GG	184 (38.41)	209 (42.14)	Reference
	Recessive model	GG	25 (5.22)	24 (4.84)	0.786	0.996 (0.968,1.025)
		GA+AA	454 (94.78)	472 (95.16)	Reference
*ARNT*					
rs10847	Genotype	TT	24 (5.01)	21 (4.23)	0.836	0.972 (0.742,1.273)
		CT	161 (33.61)	165 (33.27)	0.547	0.830 (0.452,1.523)
		CC	294 (61.38)	310 (62.50)	Reference
	Dominant model	CC	294 (61.38)	310 (62.50)	0.718	1.030 (0.877,1.209)
		CT+TT	185 (38.62)	186 (37.50)	Reference
	Recessive model	TT	24 (5.01)	21 (4.23)	0.563	0.992 (0.965,1.020)
		CT+CC	455 (94.99)	475 (95.77)	Reference
rs11204735	Genotype	CC	94 (19.62)	85 (17.14)	0.065	0.708 (0.491,1.022)
		CT	244 (50.94)	231 (46.57)	0.039	0.742 (0.558,0.986)
		TT	141 (29.44)	180 (36.29)	Reference
	Dominant model	TT	141 (29.44)	180 (36.29)	**0.023**	1.108 (1.014,1.210)
		CT+CC	338 (70.56)	316 (63.71)	Reference
	Recessive model	CC	244 (50.94)	231 (46.57)	0.173	0.918 (0.812,1.038)
		CT+TT	235 (49.06)	265 (53.43)	Reference
rs1889740	Genotype	TT	56 (11.69)	68 (13.71)	0.288	1.244 (0.832,1.861)
		CT	214 (44.68)	224 (45.16)	0.610	1.072 (0.820,1.403)
		CC	209 (43.63)	204 (41.13)	Reference
	Dominant model	CC	209 (43.63)	204 (41.13)	0.496	0.957 (0.860,1.066)
		CT+TT	270 (56.37)	292 (58.87)	Reference
	Recessive model	TT	56 (11.69)	68 (13.71)	0.344	1.023 (0.976,1.074)
		CT+CC	423 (88.31)	428 (86.29)	Reference
*AHRR*					
rs2292596	Genotype	GG	59 (12.32)	70 (14.11)	0.135	1.357 (0.910,2.024)
		GC	213 (44.47)	245 (49.40)	0.047	1.315 (1.003,1.725)
		CC	207 (43.22)	181 (36.49)	Reference
	Dominant model	CC	207 (43.22)	181 (36.49)	**0.032**	0.894 (0.807,0.991)
		GC+GG	272 (56.78)	315 (63.51)	Reference
	Recessive model	GG	59 (12.32)	70 (14.11)	0.408	1.021 (0.972,1.072)
		GC+CC	420 (87.68)	426 (85.89)	Reference
rs2672725	Genotype	GG	132 (27.56)	104 (20.97)	**0.011**	0.632 (0.444,0.900)
		GC	229 (47.81)	245 (49.40)	0.323	0.859 (0.635,1.161)
		CC	118 (24.63)	147 (29.64)	Reference
	Dominant model	CC	118 (24.63)	147 (29.64)	0.079	1.071 (0.992,1.156)
		GC+GG	361 (75.37)	349 (70.36)	Reference
	Recessive model	GG	132 (27.56)	104 (20.97)	**0.016**	0.917 (0.853,0.985)
		GC+CC	347 (72.44)	392 (79.03)	Reference
rs349583	Genotypes	GG	112 (23.38)	126 (25.40)	0.295	1.209 (0.847,1.726)
		GA	238 (49.69)	250 (50.40)	0.436	1.129 (0.832,1.533)
		AA	129 (26.93)	120 (24.19)	Reference
	Dominant model	AA	129 (26.93)	120 (24.19)	0.327	0.964 (0.895,1.038)
		GA+GG	350 (73.07)	376 (75.81)	Reference
	Recessive model	GG	112 (23.38)	126 (25.40)	0.463	1.027 (0.956,1.103)
		GA+GG	367 (76.62)	370 (74.60)	Reference

Bold value means P < 0.05.

**Table 3:** Allele frequencies of *AHR, ARNT, AHRR* genes in RA patients and controls.

SNP	Allele	RA patients	Controls	*P* value	*OR* (95% *CI*)
*AHR*					
rs2066853	A	361 (37.68)	370 (37.30)	0.861	0.994 (0.928,1.065)
	G	597 (62.32)	622 (62.70)	Reference
rs2282885	G	209 (21.82)	233 (23.49)	0.378	0.929 (0.788,1.095)
	A	749 (78.18)	759 (76.51)	Reference
*ARNT*					
rs10847	T	209 (21.82)	207 (20.87)	0.609	0.988 (0.943,1.035)
	C	749 (78.18)	785 (79.13)	Reference
rs11204735	C	432 (45.09)	401 (40.42)	**0.037**	0.922 (0.853,0.995)
	T	526 (54.91)	591 (59.58)	Reference
rs1889740	T	326 (34.03)	360 (36.29)	0.296	1.035 (0.970,1.105)
	C	632 (65.97)	632 (63.71)	Reference
*AHRR*					
rs2292596	G	331 (34.55)	385 (38.81)	0.051	1.070 (1.000,1.144)
	C	627 (65.45)	607 (61.19)	Reference
rs2672725	G	493 (51.46)	453 (45.67)	**0.010**	0.893 (0.819,0.974)
	C	465 (48.54)	539 (54.33)	Reference
rs349583	G	462 (48.23)	502 (50.60)	0.293	1.048 (0.960,1.144)
	A	496 (51.77)	490 (49.40)	Reference

Bold value means P < 0.05.

### Association of *AHR*, *ARNT*, *AHRR* Polymorphisms With RA Susceptibility

The genotype frequencies of *AHR* rs2066853, rs2282885 polymorphism were similar between RA patients and healthy controls with no significant association, moreover there was no significant difference in allele distributions of the *AHR* rs2066853, rs2282885 between RA patients and healthy controls. In the *ARNT* gene, we determined that the rs11204735 C allele frequency was significantly increased in RA patients when compared to healthy controls (C versus T: *P* = 0.037), while rs11204735 polymorphism was found to have a decreased risk of RA under the dominant model (TT versus CT+CC: *P* = 0.023). The rs11204735 CT genotype frequency was also increased in RA patients, while the significant association disappeared after multiple testing by Bonferroni correction (CT versus TT: *P* > 0.0167). In addition, no significant association was identified with *AHR* rs10847, rs1889740 in RA patients.

When investigating the genotype and allele frequencies of rs2292596, rs2672725, rs349583 in *AHRR*, we demonstrated that the rs2292596 polymorphism was significantly associated with an increased risk of RA under dominant model (CC versus GC+GG: *P* = 0.032). The rs2292596 GC genotype frequency appeared to be lower in RA patients than that in healthy controls, however, the difference was not statistically significant by Bonferroni correction (GC versus CC: *P* > 0.0167). The results also showed that rs2672725 GG genotype, G allele frequencies in RA patients were significantly higher than the healthy controls (GG versus CC: *P* = 0.011, G versus C: *P* = 0.010). Moreover, an increased risk of rs2672725 was found under the recessive model (GG versus GC+CC: *P* = 0.016). However, there was no significant difference in genotype, allele distributions of *AHRR* rs349583 between RA patients and healthy controls.

Our study also analyzed the potential association between *AHR*, *ARNT*, *AHRR* genes polymorphisms and anti-CCP, RF status in RA patients (**Table S2**). None of the SNPs exhibited significant differences between anti-CCP-positive RA patients and anti-CCP-negative RA patients, as well as RA patients with RF-positive and with RF-negative.

### Haplotype Analysis

This study constructed the haplotypes of *AHR*, *ARNT*, *AHRR* genes by SHEsis software, and three main haplotypes (AA, GA, GG) for *AHR* gene, four main haplotypes (CCC, CTC, CTT, TCC) for *ARNT* gene, six main haplotypes (CCA, CCG, CGA, CGG, GCA, GCG) for *AHRR* gene were detected by SHEsis **(** **Table 4** **)**. We found that the *ARNT* CCC haplotype frequency was significantly increased in RA patients when compared to healthy controls (*P* = 0.048).

**Table 4:** Haplotype analysis of *AHR, ARNT, AHRR* genes in RA patients and controls.

Haplotype	RA patients [n (%)]	Controls [n (%)]	*P* value	*OR* (95% CI)
*AHR* rs2066853-rs2282885				
AA	359.32 (37.5)	368.22 (37.1)	0.860	1.017 (0.846,1.222)
GA	389.68 (40.7)	390.78 (39.4)	0.563	1.055 (0.880,1.266)
GG	207.32 (21.6)	231.22 (23.3)	0.378	0.909 (0.734,1.124)
*ARNT* rs10847-rs11204735-rs1889740				
CCC	223.01 (23.3)	193.93 (19.5)	**0.048**	1.245 (1.002,1.546)
CTC	200.00 (20.9)	231.07 (23.3)	0.189	0.866 (0.699,1.073)
CTT	326.00 (34.0)	358.75 (36.2)	0.304	0.907 (0.753,1.093)
TCC	208.99 (21.8)	205.82 (20.7)	0.584	1.063 (0.855,1.320)
*AHRR* rs2292596-rs2672725-rs349583				
CCA	91.77 (9.6)	98.30 (9.9)	0.800	0.962 (0.713,1.298)
CCG	42.23 (4.4)	56.90 (5.7)	0.180	0.757 (0.503,1.139)
CGA	352.54 (36.8)	331.77 (33.4)	0.125	1.157 (0.960,1.393)
CGG	140.46 (14.7)	120.03 (12.1)	0.099	1.246 (0.959,1.619)
GCA	51.69 (5.4)	59.92 (6.0)	0.535	0.886 (0.604,1.300)
GCG	279.31 (29.2)	323.88 (32.6)	0.092	0.847 (0.699,1.027)

Frequency < 0.03 in both controls and RA patients has been dropped.

Bold value means P < 0.05.

### The Methylation Levels of *AHR*, *ARNT*, *AHRR* Genes in RA Patients and Healthy Controls

We enrolled 122 RA patients and 123 healthy controls from the subjects in the genotyping experiment for detecting methylation. The RA group included 100 females and 22 males, with an average age of 52.61 ± 13.05 years, and the control group included 82 females and 41 males with an average age of 46.93 ± 14.29 years. The final aspect of this study was to measure the methylation levels of two specific sites in *AHR* gene, two specific sites in *ARNT* gene, and a specific site in *AHRR* gene. As shown in **Table 5**, AHR_2 methylation level significantly increased in RA patients (*P* = 0.012), while AHRR_1 methylation level was significantly lower (*P* = 0.049), when compared to controls.

**Table 5:** Methylation levels of specific sites between RA patients and controls.

Specific sites	RA patients	Controls	*P* value
AHR_1	0.0076 ± 0.0008	0.0075 ± 0.0009	0.177
AHR_2	0.0075 ± 0.0007	0.0072 ± 0.0009	**0.012**
ARNT_1	0.0145 ± 0.0030	0.0144 ± 0.0029	0.671
ARNT_2	0.0067 ± 0.0013	0.0066 ± 0.0014	0.584
AHRR_1	0.9632 ± 0.0058	0.9644 ± 0.0038	**0.049**

Bold value means P < 0.05.

**Figure 1:**
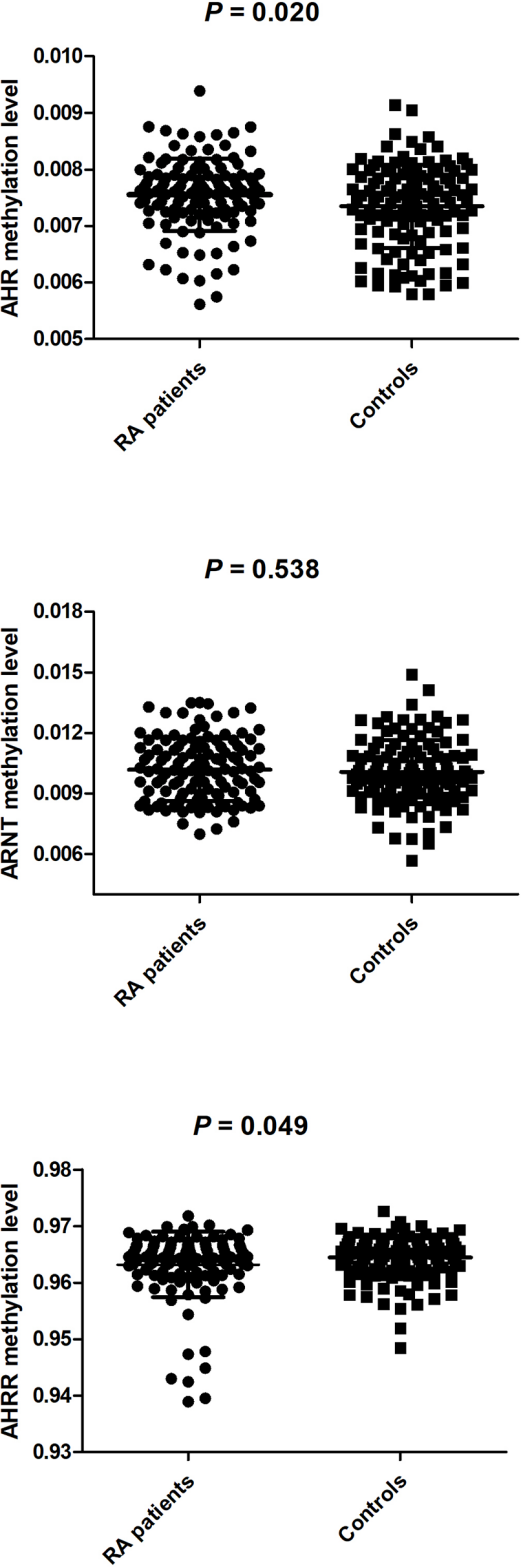
The methyation levels of AHR, ARNT, AHRR between RA patients and controls.

We further determined the cumulative methylation levels of each gene by calculating the mean methylation levels of all CpG sites on the included specific sites. The results revealed that the methylation level of AHR gene was significantly higher in RA patients (0.0076 ± 0.0006) than the healthy controls (0.0073 ± 0.0007) (*P* = 0.020). Our study only examined methylation levels of a specific site in the *AHRR* gene. Consistent with AHRR_1, AHRR methylation level was abnormally reduced in RA patients (0.9632 ± 0.0058) when compared to healthy controls (0.9644 ± 0.0038) (*P* = 0.049). There was no significant difference in ARNT methylation level between RA patients (0.0102 ± 0.0015) and healthy controls (0.0101 ± 0.0016) (*P* = 0.538) (**Figure 1**). The results also suggested that ARNT methylation level was significantly increased in anti-CCP-positive RA patients (0.0104 ± 0.0015) in comparison to anti-CCP-negative RA patients (0.0090 ± 0.0013) (*P* = 0.002). In addition, ARNT_1 methylation level in anti-CCP-positive RA patients than that in anti-CCP-negative RA patients (0.0116 ± 0.0024) (*P* = 0.002). However, AHR, AHRR methylation levels were not associated with anti-CCP, RF in RA patients (**Table 6**).

**Table 6:** Relationship between AHR, ARNT, AHRR methylation levels and anti-CCP, RF in RA patients.

Group	Anti-CCP		*P* value	RF		*P* value
	Positive (n=88)	Negative (n=14)		Positive (n=99)	Negative (n=17)	
AHR methylation level	0.0075 ± 0.0007	0.0074 ± 0.0008	0.588	0.0075 ± 0.0006	0.0075 ± 0.0007	0.962
AHR_1 methylation level	0.0076 ± 0.0008	0.0077 ± 0.0011	0.606	0.0076 ± 0.0008	0.0077 ± 0.0007	0.813
AHR_2 methylation level	0.0075 ± 0.0007	0.0072 ± 0.0008	0.689	0.0075 ± 0.0007	0.0074 ± 0.0007	0.739
ARNT methylation level	0.0104 ± 0.0015	0.0090 ± 0.0013	**0.002**	0.0102 ± 0.0015	0.0101 ± 0.0018	0.770
ARNT_1 methylation level	0.0150 ± 0.0029	0.0116 ± 0.0024	**<0.001**	0.0145 ± 0.0028	0.0145 ± 0.0039	0.915
ARNT_2 methylation level	0.0067 ± 0.0013	0.0069 ± 0.0013	0.624	0.0067 ± 0.0014	0.0065 ± 0.0009	0.424
AHRR methylation level	0.9625 ± 0.0065	0.9634 ± 0.0032	0.604	0.9627 ± 0.0062	0.9649 ± 0.0024	0.154

Bold value means P < 0.05.

### Associations Between *AHR*, *ARNT*, *AHRR* Genes Polymorphisms With Their Methylation Levels in RA Patients

This study explored the associations between *AHR*, *ARNT*, *AHRR* genes methylation levels and their different genotype in 122 RA patients (**Table 7**). The results demonstrated a significant difference in methylation level of AHRR among different genotypes of AHRR rs2672725 polymorphism in RA patients (*P* = 0.012). After multiple comparisons, the AHRR methylation level in RA patients with rs2672725 GC (0.9615 ± 0.0076) was significantly lower than RA patients with rs2672725 CC (0.9649 ± 0.0026) (*P* < 0.0167). No statistical difference in the methylation level of *AHR*, *ARNT* in RA patients with their disparate genotypes was recorded (**Table 7**).

**Table 7:** Associations between *AHR*, *ARNT*, *AHRR* genes polymorphisms with their methylation levels in RA patients.

AHR SNP	Genotype	N	AHR methylation level	P value
rs2066853	AA	17	0.0077 ± 0.0006	0.988
	GA	61	0.0075 ± 0.0006	
	GG	44	0.0076 ± 0.0007	
rs2282885	GG	8	0.0077 ± 0.0007	0.670
	GA	38	0.0076 ± 0.0005	
	AA	76	0.0075 ± 0.0007	
*ARNT* SNP	Genotype	N	ARNT methylation level	*P* value
rs10847	TT	5	0.0102 ± 0.0010	0.964
	CT	42	0.0102 ± 0.0016	
	CC	75	0.0101 ± 0.0015	
rs1889740	TT	17	0.0101 ± 0.0014	0.724
	CT	55	0.0101 ± 0.0015	
	CC	50	0.0103 ± 0.0017	
rs11204735	CC	20	0.0096 ± 0.0014	0.140
	CT	68	0.0102 ± 0.0017	
	TT	34	0.0104 ± 0.0013	
*AHRR* SNP	Genotype	N	*AHRR* methylation level	*P* value
rs2292596	GG	13	0.9651 ± 0.0017	0.063
	GC	61	0.9620 ± 0.0073	
	CC	48	0.9642 ± 0.0037	
rs2672725	GG	37	0.9642 ± 0.0039	**0.012**
	GC	53	0.9615 ± 0.0076	
	CC	32	0.9649 ± 0.0026	
rs349583	GG	35	0.9640 ± 0.0041	0.523
	GA	63	0.9627 ± 0.0059	
	AA	24	0.9634 ± 0.0076	

Bold value means P < 0.05.

## Discussion

The impact of environmental factors on the pathogenesis of autoimmune diseases has attracted increasing attention (20). AHR is considered to be a crucial regulator of host-environment interactions, especially in immune and inflammatory responses (21, 22). Proper regulation of AHR-mediated signal transduction might be critical for maintaining immune cell function in autoimmunity, while abnormal AHR signaling has been closely related to the pathogenesis of autoimmune diseases (13). Clarifying the role of AHR in disease and health is crucial in understanding the disease occurrence process and individual differences in treatment response, both of which might contribute to the development of novel therapies (23). RA is a common autoimmune disease and AHR plays a role in regulating the response to the environment factors and may contribute to disease development. Clinical studies had demonstrated that AHR expression was approximately two-fold higher in RA patients than in controls (24). AHRR expression of the synovia was significantly increased from RA patients who smoke cigarettes, but not from the patients who do not smoke, which indicated that there was a potential interaction between AHR activation and cigarette smoking in RA patients (25). The murine models of RA suggested that AHR activation with 2,3,7,8-tetrachlorodibenzo-p-dioxin (TCDD) could contribute to RA disease severity, disease progression, osteoclasts differentiation, and increase the numbers of IL17-expressing cells in the inflamed joints (10). In addition, previous studies had shown that several SNPs located near AHR-regulated genes might contribute to AHR-dependent disease mechanisms (26). Therefore, we investigated the association between multiple SNPs in AHR signaling pathway genes (*AHR*, *ARNT*, *AHRR*) and RA genetic risk, and detected the methylation levels of these genes in RA patients.

A large number of studies had discussed the association of the genetic polymorphism of *AHR*, *ARNT*, *AHRR* genes and human diseases such as Crohn’s disease (CD), lung cancer, male infertility, and autoimmune diseases (27–30). Zorlu et al. evaluated the relationship between multiple SNPs in the AHR signaling pathway genes, including *AHR*, *ARNT*, *AHRR* and MS susceptibility, and their results inferred that the AHR signaling pathway genetic variation might contribute to MS pathogenesis (14). However, our study did not indicate a significant association of *AHR* rs2066853, rs2282885 polymorphism with RA susceptibility, and rs2158041 was excluded as it did not satisfy HWE. Consistent with our findings, Cheng et al. demonstrated that rs2066853 polymorphism did not have any effect on RA susceptibility in Han Chinese populations (31). These results suggested that *AHR* gene variation might not be associated with RA, however, other functional SNPs required further investigation.

Our study also determined that *ARNT* rs11204735 polymorphism was significantly associated with an increased risk of RA, and that a decreased risk of rs11204735 polymorphism was observed under the dominant model. This result was similar to Schurman’s study (30), and further reflected the importance of rs11204735 polymorphism in the pathogenesis of autoimmune diseases. Haplotype analysis demonstrated that *ARNT* CCC haplotype frequency significantly increased in RA patients in comparison to controls. Therefore, this present study provided additional evidence that *ARNT* gene was an important susceptibility gene in RA, and reproducibility studies were needed to verify our results. A previous study demonstrated that rs2292596 genotype frequency was significant with genetic susceptibility to RA patients (31). Their study also demonstrated that the rs2292596 G allele might have a dangerous effect on RA (31), while we identified an increased frequency of rs2292596 G allele with no statistical significance. This difference might be due to sample size and experimental methods. Our result was also the first to show that *AHRR* rs2672725 GG genotype, G allele frequencies in RA patients were significantly increased, and that this SNP could be a novel susceptibility locus to RA.

In addition to DNA sequence, epigenetic variations also contained important genetic information, and the key role of epigenetic variation in the pathogenesis of human disease could not be ignored (32, 33). For example, DNA hypomethylation was associated with differentiation and proliferation of inflammatory processes, resulting in the increased transcription and secretion of inflammatory proteins (34). At present, studying the role of DNA methylation in autoimmune diseases offers an interesting perspective. One study detected the methylation status of lymphocytes in patients with systemic lupus erythematosus, RA, and found a significant hypomethylation in T cells (35). Due to the possible role of *AHR*, *ARNT*, *AHRR* genes in RA pathogenesis, we also examined the methylation status of these genes in RA patients. The results revealed that AHR methylation level was significantly increased, while AHRR methylation level was abnormally reduced in RA patients. These data confirmed that *AHR*, *AHRR* genes were involved in the development of RA, and provided valuable information for further revealing RA pathogenesis. RA patients could be divided into different subgroups according to the status of autoantibodies, including RF and anti-CCP, which was of great significance when selecting appropriate treatments. Our study also determined that ARNT methylation level was related to the anti-CCP status of RA patients. In addition, we also revealed that *AHRR* rs2672725 polymorphism was significantly associated with *AHRR* methylation level in RA patients. We assumed that rs2672725 might be involved in the pathogenesis of RA by affecting *AHRR* methylation level, however further verification was needed.

Our study had several limitations. First, the sample size was insufficient and might affect the power of this study. Second, this study failed to analyze the influence of the interaction between environmental factors and gene variation on RA occurrence due to a lack of information on environmental factors. Undoubtedly, a complete understanding of the precise role of AHR signaling pathway genes in RA pathogenesis will require additional experiments and clinical studies with larger sample sizes.

In conclusion, our results provided strong evidence that the *ARNT* gene rs11204735, *AHRR* gene rs2292596, rs2672725 polymorphisms were related to RA susceptibility in the Chinese population, while *AHR* genetic variation might not be associated with RA risk. These three SNPs might modify individual genetic susceptibility to RA, which could provide a basis for early detection of RA susceptible individuals. Furthermore, an increased methylation level of *AHR*, as well as a decreased methylation level of *AHRR*, was identified in RA patients, and ARNT methylation level was related to anti-CCP in RA patients. We can assume that AHR, AHRR methylation level might be used as novel auxiliary biomarkers for RA and ARNT methylation level could be used to distinguish the different serotypes of RA.

## Data Availability Statement

The data presented in the study are deposited in the dbSNP repository, accession number 1063327. Further inquiries can be directed to the corresponding authors.

## Ethics Statement

The studies involving human participants were reviewed and approved by the Ethical Committee of the First Affiliated Hospital of USTC (Hefei, Anhui, China). The patients/participants provided their written informed consent to participate in this study.

## Author Contributions

X-ML and G-SW designed the study. T-PZ and H-ML conducted the experiment. RL performed the statistical analyses. H-ML performed the statistical analyses. NX and ZT participated in the collection of samples. T-PZ drafted the manuscript. X-ML, G-SW and RL contributed to manuscript revision. All authors contributed to the article and approved the submitted version.

## Funding

This work was supported by the Fundamental Research Funds for the Central Universities (WK9110000180, WK9110000148), Anhui Provincial Natural Science Foundation (2108085QH362), and National Natural Science Foundation of China (81871271).

## Conflict of Interest

The authors declare that the research was conducted in the absence of any commercial or financial relationships that could be construed as a potential conflict of interest.

## Publisher’s Note

All claims expressed in this article are solely those of the authors and do not necessarily represent those of their affiliated organizations, or those of the publisher, the editors and the reviewers. Any product that may be evaluated in this article, or claim that may be made by its manufacturer, is not guaranteed or endorsed by the publisher.
