# Supplementary material for: The Contribution of Genetic Variation and Aberrant Methylation of Aryl Hydrocarbon Receptor Signaling Pathway Genes to Rheumatoid Arthritis
Source: Front Immunol. 2022 Mar 2;13:823863. doi: 10.3389/fimmu.2022.823863 (PMC8924038; doi:10.3389/fimmu.2022.823863)
Supplement: Supplementary file 1 [file Table_1.doc]

**Table S1** The primers of specific sites in *AHR*, *ARNT*, *AHRR* genes

| Gene | Fragment | Forward primer | Reverse primer |
| --- | --- | --- | --- |
| AHR | AHR_1 | TTTTTGGGTGAGGGAGGTG | CCRCCAACTCAAAACAAAAACAA |
|  | AHR_2 | GGAAGTYGGTGTAGAAAAYGTGAG | CCTCTATCTCCCAACCRACCT |
| ARNT | ARNT_1 | AGGGTTATTTATTGYGTTAGTTAGTTTAGTG | CRAACCCCTAACCACAAATAATC |
|  | ARNT_2 | GGAGGGAGGGGAAAAGAAA | CCCTCCCTTCACTAAACTAACTAAC |
| AHRR | AHRR _1 | AAATATATAYGGAGAGATGTGAAAAGGT | TTCAAACACRTAAACTCCCTTTAATC |
