# Supplementary material for: The Contribution of Genetic Variation and Aberrant Methylation of Aryl Hydrocarbon Receptor Signaling Pathway Genes to Rheumatoid Arthritis
Source: Front Immunol. 2022 Mar 2;13:823863. doi: 10.3389/fimmu.2022.823863 (PMC8924038; doi:10.3389/fimmu.2022.823863)
Supplement: Supplementary file 2 [file Table_2.doc]

**Table S2** Associations between *AHR, ARNT, AHRR* genes polymorphisms and anti-CCP, RF in RA patients

| SNP | Allele | Clinical features | Group | Genotype | | | *P* value | Allele | | *P* value |
| --- | --- | --- | --- | --- | --- | --- | --- | --- | --- | --- |
|  | (M/m) |  |  | MM | Mm | mm |  | M | m |  |
| *AHR* | | | | | | | |  | | |
| rs2066853 | G/A | anti-CCP | Positive | 138(39.09) | 172(48.73) | 43(12.18) | 0.085 | 448(63.46) | 258(36.54) | 0.101 |
|  |  |  | Negative | 27(34.62) | 34(43.59) | 17(21.79) |  | 88(56.41) | 68(43.59) |  |
|  |  | RF | Positive | 151(39.74) | 181(47.63) | 48(12.63) | 0.166 | 483(63.55) | 277(36.45) | 0.094 |
|  |  |  | Negative | 26(33.33) | 36(46.15) | 16(20.51) |  | 88(56.41) | 68(43.59) |  |
| rs2282885 | A/G | anti-CCP | Positive | 214(60.62) | 123(34.84) | 16(4.53) | 0.735 | 551(78.05) | 155(21.95) | 0.896 |
|  |  |  | Negative | 48(61.54) | 25(32.05) | 5(6.41) |  | 121(77.56) | 35(22.44) |  |
|  |  | RF | Positive | 225(59.21) | 133(35) | 22(5.79) | 0.262 | 583(76.71) | 177(23.29) | 0.102 |
|  |  |  | Negative | 53(67.95) | 23(29.49) | 2(2.56) |  | 129(82.69) | 27(17.31) |  |
| *ARNT* | | | | | | | | | | |
| rs10847 | C/T | anti-CCP | Positive | 218(61.76) | 116(32.86) | 19(5.38) | 0.638 | 552(78.19) | 154(21.81) | 0.489 |
|  |  |  | Negative | 44(56.41) | 30(38.46) | 4(5.13) |  | 118(75.64) | 38(24.36) |  |
|  |  | RF | Positive | 232(61.05) | 126(33.16) | 22(5.79) | 0.329 | 590(77.63) | 170(22.37) | 0.985 |
|  |  |  | Negative | 45(57.69) | 31(39.74) | 2(2.56) |  | 121(77.56) | 35(22.44) |  |
| rs11204735 | T/C | anti-CCP | Positive | 106(30.03) | 174(49.29) | 73(20.68) | 0.841 | 386(54.67) | 320(45.33) | 0.739 |
|  |  |  | Negative | 21(26.92) | 41(52.56) | 16(20.51) |  | 83(53.21) | 73(46.79) |  |
|  |  | RF | Positive | 113(29.74) | 191(50.26) | 76(20.00) | 0.424 | 417(54.87) | 343(45.13) | 0.501 |
|  |  |  | Negative | 18(23.08) | 45(57.69) | 15(19.23) |  | 81(51.92) | 75(48.08) |  |
| rs1889740 | C/T | anti-CCP | Positive | 152(43.06) | 164(46.46) | 37(10.48) | 0.513 | 468(66.29) | 238(33.71) | 0.389 |
|  |  |  | Negative | 39(50.00) | 31(39.74) | 8(10.26) |  | 109(69.87) | 47(30.13) |  |
|  |  | RF | Positive | 165(43.42) | 175(46.05) | 40(10.53) | 0.973 | 505(66.45) | 255(33.55) | 0.836 |
|  |  |  | Negative | 35(44.87) | 35(44.87) | 8(10.26) |  | 105(67.31) | 51(32.69) |  |
| *AHRR* | | | | | | | | | | |
| rs2292596 | C/G | anti-CCP | Positive | 148(41.93) | 164(46.46) | 41(11.61) | 0.612 | 460(65.16) | 246(34.84) | 0.688 |
|  |  |  | Negative | 33(42.31) | 33(42.31) | 12(15.38) |  | 99(63.46) | 57(36.54) |  |
|  |  | RF | Positive | 164(43.16) | 170(44.74) | 46(12.11) | 0.810 | 498(65.53) | 262(34.47) | 0.734 |
|  |  |  | Negative | 31(39.74) | 38(48.72) | 9(11.54) |  | 100(64.10) | 56(35.90) |  |
| rs2672725 | C/G | anti-CCP | Positive | 87(24.65) | 179(50.71) | 87(24.65) | 0.591 | 353(50.00) | 353(50.00) | 0.664 |
|  |  |  | Negative | 20(25.64) | 35(44.87) | 23(29.49) |  | 75(48.08) | 81(51.92) |  |
|  |  | RF | Positive | 103(27.11) | 181(47.63) | 96(25.26) | 0.729 | 387(50.92) | 373(49.08) | 0.949 |
|  |  |  | Negative | 19(24.36) | 41(52.56) | 18(23.08) |  | 79(50.64) | 77(49.36) |  |
| rs349583 | A/G | anti-CCP | Positive | 90(25.50) | 176(49.86) | 87(24.65) | 0.564 | 356(50.42) | 350(49.58) | 0.288 |
|  |  |  | Negative | 24(30.77) | 38(48.72) | 16(20.51) |  | 86(55.13) | 70(44.87) |  |
|  |  | RF | Positive | 98(25.79) | 194(51.05) | 88(23.16) | 0.695 | 390(51.32) | 370(48.68) | 0.765 |
|  |  |  | Negative | 21(26.92) | 36(46.15) | 21(26.92) |  | 78(50.00) | 78(50.00) |  |
